# Ferroptosis in Cardiovascular Disease and Cardiomyopathies: Therapeutic Implications of Glutathione and Iron Chelating Agents

**DOI:** 10.3390/biomedicines12030558

**Published:** 2024-03-01

**Authors:** John Dawi, Scarlet Affa, Edgar Gonzalez, Yura Misakyan, David Nikoghosyan, Karim Hajjar, Samuel Kades, Sabrina Fardeheb, Hayk Mirzoyan, Vishwanath Venketaraman

**Affiliations:** 1 College of Osteopathic Medicine of the Pacific, Western University of Health Sciences, Pomona, CA 91766, USA; john.dawi@westernu.edu (J.D.); edgar.gonzalez@westernu.edu (E.G.); yura.misakyan@westernu.edu (Y.M.); david.nikoghosyan@westernu.edu (D.N.); karim.hajjar@westernu.edu (K.H.); samuel.kades@westernu.edu (S.K.); sabrina.fardeheb@westernu.edu (S.F.); 2 Department of Chemistry, Physics, and Engineering, Los Angeles Valley College, Valley Glen, CA 91401, USA; 3 College of Osteopathic Medicine, Touro University Nevada, Henderson, NV 89014, USA; hmirzoya@student.touro.edu

**Keywords:** ferroptosis, GPX4, NRF2, GSH, oxidative stress, cardiomyopathy, iron chelating agents, flavonoids

## Abstract

This review explores ferroptosis, a form of regulated cell death reliant on iron-induced phospholipid peroxidation, in diverse physiological and pathological contexts, including neurodegenerative disorders, and ischemia-reperfusion. In the realm of cardiovascular diseases, it significantly contributes to cardiomyopathies, including dilated cardiomyopathy, hypertrophic cardiomyopathy, and restrictive cardiomyopathy. Ferroptosis involves intricate interactions within cellular iron metabolism, lipid peroxidation, and the balance between polyunsaturated and monounsaturated fatty acids. Molecularly, factors like p53 and NRF2 impact cellular susceptibility to ferroptosis under oxidative stress. Understanding ferroptosis is vital in cardiomyopathies, where cardiac myocytes heavily depend on aerobic respiration, with iron playing a pivotal role. Dysregulation of the antioxidant enzyme GPX4 is linked to cardiomyopathies, emphasizing its significance. Ferroptosis’s role in myocardial ischemia-reperfusion injury, exacerbated in diabetes, underscores its relevance in cardiovascular conditions. This review explores the connection between ferroptosis, the NRF2 pathway, and atherosclerosis, emphasizing their roles in protecting cells from oxidative stress and maintaining iron balance. It discusses the use of iron chelating agents in managing iron overload conditions, with associated benefits and challenges. Finally, it highlights the importance of exploring therapeutic strategies that enhance the glutathione (GSH) system and the potential of natural compounds like quercetin, terpenoids, and phenolic acids in reducing oxidative stress.

## 1. Introduction to Ferroptosis

Cellular destruction is a dynamic process in which cells perpetually capitalize. The degradation of their complex structure allows for cellular genesis. In 2012, a newly identified cell death pathway was discovered, initially coined “ferroptosis”. Ferroptosis is a form of regulated cell death dissimilar from apoptosis, necrosis, and autophagy. Its unique process encompasses an iron-dependent phospholipid peroxidation for which its regulation is metabolically multifactorial, specifically dabbling in the realm of redox homeostasis, iron handling, mitochondrial activity, and metabolism of amino acids, lipids, and sugars, including but not limited to other various signaling pathways relevant to disease [1]. In broad terms, lipid peroxidation is characterized as a phenomenon in which oxidizing agents, including free radicals or nonradical species, target lipids containing carbon–carbon double bonds, particularly polyunsaturated fatty acids (PUFAs). This process involves the abstraction of hydrogen from carbon, leading to the insertion of oxygen and the formation of lipid peroxyl radicals and hydroperoxides, as previously outlined [1]. Lipid peroxides represent the outcome of the oxidation process affecting phospholipids and polyunsaturated fatty acids [1].

Ferroptosis is a significant biological process crucial in various physiological and pathological situations. It has been implicated in several diseases, such as neurodegenerative disorders, cancer, and ischemia-reperfusion [2,3]. In terms of cardiovascular disease (CVD) and cardiomyopathies, CVD remains the foremost cause of disease burden and premature death globally [4,5]. Data from 2019 highlighted an alarming prevalence of up to 523 million CVD cases, resulting in 18.6 million deaths, primarily attributed to ischemic heart disease (49%) and stroke (17.7%) [6]. Over the past few decades, researchers have implicated various forms of regulated and unregulated cell death in cardiac and vascular cells in the pathogenesis of diverse heart diseases, including myocardial infarction (MI), heart failure of different origins, myocarditis, and congenital heart disease [7,8,9]. Among these, ferroptosis emerges as a distinctive form of iron-dependent, non-apoptotic cell death, characterized by the accumulation of lipid hydroperoxides that cause oxidative damage to cell membranes [10]. Morphologically and mechanistically, ferroptosis stands apart from other forms of regulated cell death [11]. Recent studies have proposed that ferroptosis plays a role in the demise of cardiac and vascular cells induced by various stressors [12,13,14]. Consequently, ferroptosis holds significant implications in the mechanisms underlying CVDs and presents a promising target for preventive approaches [15,16]. By comprehending ferroptosis’s molecular mechanisms and regulatory pathways, researchers may gain valuable insights to develop targeted therapeutic strategies for such specific diseases.

**Figure 1:**
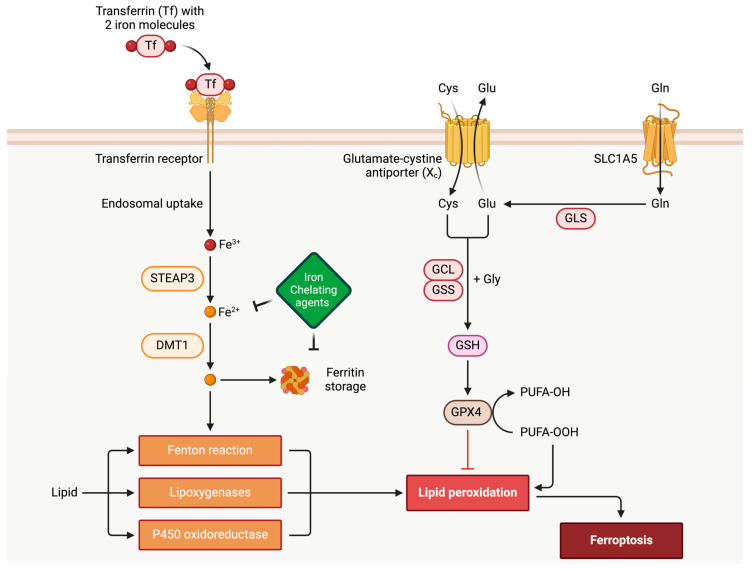
The process of ferroptosis. The overview of ferroptosis, as depicted in the accompanying figure, highlights its essential components. It begins with the metabolic generation of ROS, serving as key substrates for phospholipid peroxidation. The figure above portrays iron-dependent reactions crucial for initiating and propagating phospholipid peroxidation, integral to ferroptosis progression. It also emphasizes the role of surveillance mechanisms, particularly GPX4, in regulating and containing phospholipid peroxidation within this complex biological pathway. Furthermore, the figure demonstrates the inhibitory influence of GSH and iron chelating agents on iron-dependent ROS generation. The SLC1A5 transporter regulates the rate-limiting step of glutamine uptake, which is catalyzed and broken down into glutamate through GLS (glutaminase) as a part of GSH generation. STEAP3 and DMT1 are endosomal proteins. The complex of transferrin with Tfr1 is internalized into the endosomal compartment. Iron is released from the complex with transferrin inside endosomes, and then iron is transported to the cytosol by DMT1.

Ferroptosis is a tightly regulated process involving various cellular components and signaling pathways. A key feature of ferroptosis is the aggregation of lipid peroxides, which consists of reactive oxygen species (ROS) generated by the oxidation of polyunsaturated fatty acids (PUFAs) in cellular membranes, for which its process is driven by iron-dependent reactions, explicitly involving the Fenton reaction (Figure 1). PUFAs play a vital role as essential components of cell membranes at the structural level. Extensive lipid peroxidation can significantly alter the chemical and geometric properties of the lipid bilayer. Moreover, the buildup of peroxidative lipids can lead to the formation of membrane pores, compromising the barrier function of the membrane. This, in turn, reduces membrane thickness and alters membrane permeability. It is acknowledged that the enzyme GPX4 tightly mediates the ferroptosis surveillance mechanism. This enzyme catalyzes the conversion of lipid peroxides to their respective alcohols, preventing their accumulation and playing a protective role in cells. Glutathione is an essential cofactor for GPX4 functioning. Ultimately, exhausting the glutathione levels would decrease GPX4 activity and intensify its propensity for ferroptosis [17] (Figure 1). Glutathione peroxidase 4, also known as phospholipid hydroperoxide glutathione peroxidase (GPX4), possesses a unique capability to reduce hydroperoxides found in intricate lipids like phospholipids, cholesterol, and cholesterolester hydroperoxides, even when they are incorporated into biomembranes or lipoproteins [18]. This ability is crucial for preventing the iron (Fe^2+^)-dependent formation of harmful lipid reactive oxygen species (ROS) [18]. The significance of GPX4 extends to normal physiology, as its absence is incompatible with life due to its pivotal role in preserving mitochondrial function, inflammation, differentiation, immunity, and cell death [18]. The inhibition of GPX4 function results in lipid peroxidation and can trigger ferroptosis, an iron-dependent, non-apoptotic form of cell death [18].

Iron heavily influences ferroptosis, as it is critical in facilitating the Fenton reaction, described by H.J.H Fenton in 1894. This reaction generates highly reactive hydroxyl radicals from hydrogen peroxide and lipid hydroperoxides. When unstable iron accumulates and iron metabolism becomes dysregulated, it promotes the occurrence of ferroptosis. More specifically, the oxidation of organic substrates by iron (II) with hydrogen peroxide (H_2_O_2_) explains the dependency of ferroptosis on iron, as redox-active iron pools can directly catalyze the propagation of lipid peroxidation to form the damaging species that lead to said cell death [17]. Various enzymes and metabolic pathways tightly regulate the synthesis and availability of both PUFAs and MUFAs. Notably, acyl-CoA synthetase long-chain family member 4 (ACSL4) and lysophosphatidylcholine acyltransferase 3 (LPCAT3) play crucial roles in promoting ferroptosis by incorporating PUFAs into cellular phospholipids, especially phosphatidylethanolamine. Conversely, stearoyl-CoA desaturase (SCD) and acyl-CoA synthetase long-chain family member 3 (ACSL3)-mediated production or the activation of MUFAs hinder ferroptosis by displacing PUFAs from the cellular lipid pool. Additionally, other sources contribute to the availability of lipids for subsequent lipid peroxidation during ferroptosis. These sources include polyunsaturated ether phospholipids (PUFA-ePLs) derived from peroxisomes, fatty acids produced through lipophagy, or metabolic reprogramming from mitochondria (such as glutamine-derived anaplerotic flux or pyruvate oxidation).

Overall, the delicate balance between the availability of PUFAs and MUFAs, their incorporation into cellular lipids, and subsequent lipid peroxidation pathways dictate the susceptibility of cells to ferroptosis [19]. In specific circumstances, both p53 and NRF2 can influence the vulnerability of cells to ferroptosis, thereby impacting the cell’s fate under oxidative conditions. Its main effect is accumulating lethal lipid reactive oxygen species (ROS). This process has implications for various human diseases, and its inhibition has shown promise in reducing clinical symptoms in experimental models of ischemia-reperfusion-induced renal failure and heart injury. Additionally, ferroptosis has been found to induce tumor cell death, making small molecules like Erastin potential candidates for cancer treatments.

The tumor suppressor protein p53 responds to DNA damage by inducing apoptosis and ferroptosis. Interestingly, the inhibitor of the apoptosis-stimulating protein of p53 (iASPP) not only inhibits p53-induced apoptosis, but also supports tumor growth, contributing to chemoresistance in human cancer cells. Notably, iASPP exhibits substantial anti-ROS activity in the cytoplasm, independently of p53, promoting the accumulation and nuclear translocation of nuclear factor (erythroid-derived 2)-like 2 (Nrf2). Nrf2 is a transcription factor that confers cellular protection against oxidative stress associated with various forms of cell death, including ferroptosis, apoptosis, and autophagy. Activation of Nrf2 in the nucleus has been reported to protect against numerous diseases and multiple airway disorders, including acute lung injury (ALI) [20].

p53 exhibits the potential to impede or hinder ferroptosis. In a model of vascular calcification associated with hyperlipidemia, p53 has been observe to intriguingly enhance the expression of SLC7A11, safeguarding vascular smooth muscle cells (VSMCs) against ferroptotic cell death [21]. Periostin, on the other hand, heightens VSMC sensitivity to ferroptosis by suppressing p53, and this effect is counteracted by metformin treatment. Pre-treating cells with the MDM2 inhibitor Nutlin-3, which stabilizes p53, delays the onset of ferroptosis in cancer cells [22]. This delay is attributed to the induction of p21 by p53. However, the cell cycle arrest mediated by p21 itself does not underlie this delay. Instead, p21 induction redirects serine usage from nucleotide biogenesis to GSH synthesis, with GSH acting as an inhibitor of ROS and ferroptosis [22]. Another study indicated an inverse correlation between p21 levels and cellular sensitivity to ferroptosis induced by the ferroptosis inducers (FINs) Erastin or IKE [23]. Furthermore, p21 acts as an independent barrier to ferroptosis even in the absence of p53 [23].

In colorectal cancer cell lines SW48 and HCT116, p53 deletion heightens sensitivity to Erastin-triggered ferroptosis [24]. Mechanistically, dipeptidyl peptidase 4 (DPP4) intensifies ferroptosis in p53-deficient cells by binding NADPH oxidase 1 and enhancing ROS generation, leading to lipid peroxidation and ferroptosis. p53 directly binds and sequesters DPP4 in the nucleus, abolishing its ferroptosis-promoting activity. Mitochondrial activity contributes to ferroptosis [25]. PARK2 (Parkin), a p53 target gene mediating mitophagy, decreases the number of mitochondria and cellular sensitivity to Erastin-mediated ferroptosis [25]. Hence, p53 may restrict cysteine deprivation-induced ferroptosis by activating Parkin expression. However, conflictingly, another study reports an inhibitory effect of p53 on Parkin activity [26].

ACSL4, a pivotal regulator of membrane PUFA generation for ferroptosis [27], is post-transcriptionally downregulated by miR-34, a p53-activated microRNA (miRNA) [28]. The p53/miR-34/ACSL4 axis may suppress ferroptosis by limiting the lipid peroxidation substrate. Intriguingly, miR-34 induction partially contributes to p53-promoted apoptosis. Nonetheless, other studies have argued that p53 upregulates ACSL4 levels [28]. The precise roles of Parkin and ACSL4 in the p53-mediated ferroptosis pathway necessitate further investigation.

## 2. CVD and Cardiomyopathy Pathology and Pathophysiology

**Figure 2:**
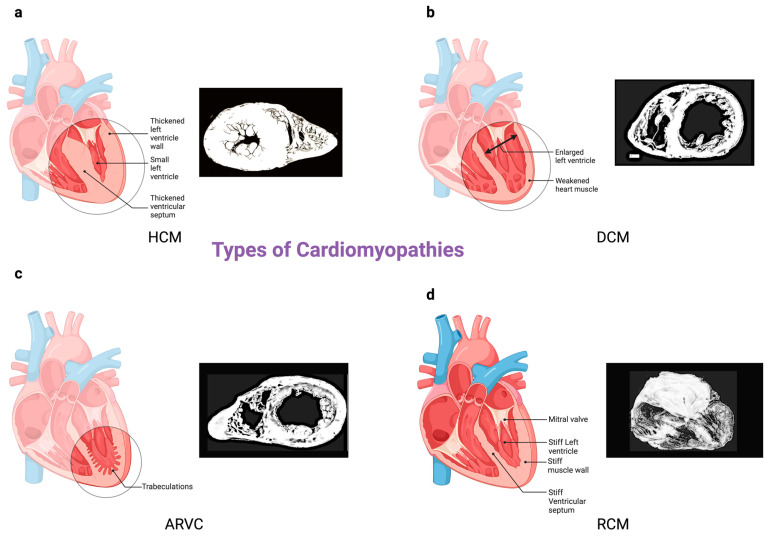
Types of Cardiomyopathies (**a**–**d**). Hypertrophic cardiomyopathy (HCM) is characterized by left-ventricular hypertrophy without chamber dilation and is primarily attributed to autosomal dominant mutations affecting genes responsible for sarcomere proteins. Dilated cardiomyopathy (DCM) is characterized by ventricular enlargement, normal left-ventricular wall thickness, and systolic dysfunction, primarily inherited in an autosomal dominant manner. Arrhythmogenic right-ventricular cardiomyopathy (ARVC) is an inherited condition associated with desmosomal protein abnormalities, leading to fibrofatty infiltration in healthy myocardium, ultimately resulting in thinning and ballooning of the ventricular wall, typically in the right ventricle. On the other hand, restrictive cardiomyopathy (RCM) is a heterogeneous condition encompassing various etiologies and is primarily defined by functional impairments rather than anatomical changes.

Cardiomyopathy is a disease that causes the heart’s muscles to struggle with pumping blood efficiently to the body. Cardiomyopathies vary in prevalence, with most of the patients falling into four major categories: hypertrophic cardiomyopathy (HCM), dilated cardiomyopathy (DCM), arrhythmogenic right ventricular cardiomyopathies (ARVC), and restrictive cardiomyopathies (RCM) (Figure 2).

**Figure 3:**
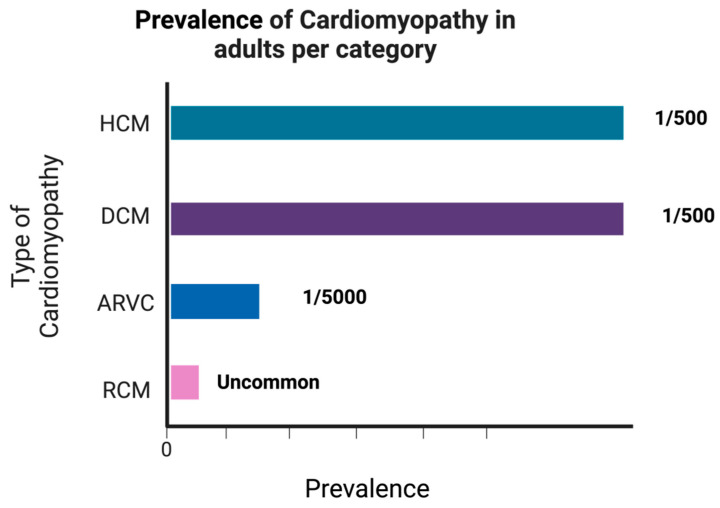
The prevalence of the four major types of cardiomyopathies. HCM and DCM are the most prevalent in the population. ARVC is the third most common condition, whereas RCV is the least common.

Dilated cardiomyopathy (DCM) is marked by ventricular enlargement, normal left-ventricular wall thickness, and systolic dysfunction, with familial cases accounting for 25% to 35% of occurrences [29] (Figure 3). DCM primarily displays an autosomal dominant inheritance pattern [29]. Symptoms such as fatigue, chest pain, edema, and shortness of breath can be vague, but it is the most common cause of heart failure. Common causes of DCM include diabetes, hypertension, pregnancy, hemochromatosis, and excessive alcohol intake. To diagnose DCM, an echocardiogram and electrocardiogram can be used. Treatment can vary based on the severity of the condition; the goal is to help the heart effectively pump blood to the rest of the body. The perseverance of DCM can be challenging to estimate as many patients go undiagnosed until untimely cardio-related mortality [30]. It has been associated with over 50 million Americans fulfilling the American Heart Association—American College of Cardiology definition of dilated cardiomyopathy [29].

Hypertrophic cardiomyopathy (HCM), on the other hand, stands as the most prevalent primary cardiomyopathy, affecting 1 in 500 individuals [29] (Figure 3). This condition is characterized by left-ventricular hypertrophy without chamber dilation and is primarily attributed to autosomal dominant mutations in genes responsible for sarcomere proteins [29]. HCM is associated with the sudden death of young athletes. The anterior leaflet of the mitral valve is pulled closer to the septum due to the location of the outflow tract and papillary muscle, which can cause mitral regurgitation and decreased ejection fraction [30,31]. The prevalence of HCM is 0.2% worldwide, and 25% of first-degree relatives of patients are diagnosed with HCM. HCM has a mortality rate of 1–4%, with a constant decline in the past years. Most cases of HCM go undetected as the first clinical presentation is sudden death. Patients with a family history of HCM and abnormally high blood pressure should undergo screening for the disorder [30].

Restrictive cardiomyopathy (RCM), representing the least common among major cardiomyopathies at 2% to 5% of cases, encompasses a spectrum of underlying etiologies and is distinguished by functional impairments rather than anatomical changes [29]. It is typified by compromised ventricular filling and maintained systolic function, stemming from heightened myocardial stiffness, and may manifest as a primary condition or secondary to various factors, including amyloidosis, sarcoidosis, radiation therapy, and scleroderma [31] (Figure 3). Risk factors include hemochromatosis, connective tissue disease, sarcoidosis, and amyloidosis. Restrictive cardiomyopathy can be treated with corticosteroids if it causes sarcoidosis, phlebotomy if the cause is hemochromatosis, and it can also be treated with diuretics, beta-blockers, and calcium channel blockers [32]. Restrictive cardiomyopathy is represented on ECG with slurred ST segment on V1-3, inverted T waves V2, and V3 with no right bundle branch block [33]. An echocardiogram will show an isolated right ventricle failure and a localized right ventricle aneurysm, i.e., arrhythmogenic right-ventricular cardiomyopathy (ARVC), with an estimated prevalence of 1 in 1000 to 5000 (Figure 3). ARVC arises from inherited anomalies in desmosome proteins, giving rise to fibrofatty infiltration in healthy myocardium. This process results in thinning and ballooning of the ventricular wall, typically within the right ventricle.

Cardiovascular disease (CVD) encompasses a complex array of disorders affecting the heart and blood vessels, with a multifaceted pathophysiology involving intricate interplays of genetic, environmental, and lifestyle factors [34]. Atherosclerosis, a hallmark feature of CVD, involves the gradual accumulation of cholesterol, lipids, and inflammatory cells in arterial walls, forming atherosclerotic plaques. These plaques may undergo rupture or erosion, triggering thrombosis and occlusion of blood vessels, leading to myocardial infarction or stroke [35,36]. Endothelial dysfunction, characterized by impaired nitric oxide bioavailability and increased vascular inflammation, plays a pivotal role in the initiation and progression of atherosclerosis [35]. Hypertension, a major risk factor for CVD, imposes excessive pressure on arterial walls, promoting vascular remodeling and atherosclerotic changes [36]. Additionally, oxidative stress and inflammation contribute significantly to CVD pathophysiology, exacerbating atherosclerotic plaque formation and destabilization [36]. Dyslipidemia, characterized by elevated levels of low-density lipoprotein cholesterol and reduced high-density lipoprotein cholesterol, further amplifies atherosclerotic processes [35]. Chronic exposure to these risk factors culminates in structural and functional changes within the heart, leading to conditions such as myocardial infarction, heart failure, and cardiomyopathies. Understanding the intricate mechanisms involved in CVD pathophysiology is crucial for developing effective preventive and therapeutic strategies.

**Figure 4:**
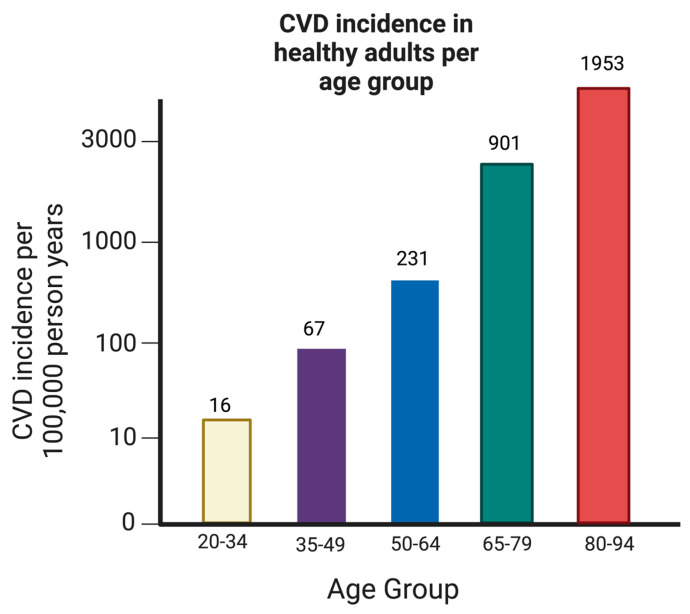
Incidence of CVD in different age groups. The prevalence of cardiovascular disease (CVD) exhibits a pronounced exponential rise in concordance with advancing age. Predominantly, individuals afflicted by CVD are aged 50 years or older, with markedly sparse occurrences observed within the demographic, spanning 20 to 34 years of age.

A longitudinal study published in 2017 (*n* = 31,856) addressed that despite the medical world’s best efforts to control patients’ BP, there were still cases of CVD. After 7.7 years of the initial study, 2584 patients had cases of CVD. With a 95% CI, 63% of those patients had a CVD event with systolic/diastolic BP of <140/90 mmHg, 58.4% of those patients were taking antihypertensive medication, and 68.1% of those patients were not taking antihypertensive medication [37,38]. The incidence of CVD increased exponentially with age, where most cases fall in the above-50-years-of-age patient cohort (Figure 4). Most of the CVD events occurred in patients with a BP of <140/90 mmHg and who were under 65 years of age [39,40]. This study concludes that higher BP measurements are often associated with CVD risk, and most of those incidents occur in US adults with BP < 140/90 mmHg [40,41]. The other risk factors remain potential reasons why CVD cases are still highly prevalent, even with medication measures.

## 3. Ferroptosis Implication in Cardiac Myocytes and Cardiovascular Disease

Cardiac myocytes, also known as cardiomyocytes or heart muscle cells, are the primary contractile cells responsible for the heart’s rhythmic pumping. These elongated, striated cells are specialized for continuous cyclic contraction and relaxation, ensuring the constant circulation of blood throughout the body. Connected through intercalated discs, the myocytes form a syncytium that allows synchronized contractions. Cardiac myocytes are also rich in mitochondria, catering to the high-energy demands of continuous contraction. Unlike many other cells that can switch between aerobic and anaerobic metabolic pathways depending on oxygen availability, cardiac myocytes rely almost exclusively on aerobic respiration to meet their energy needs. Anaerobic metabolism plays a role in maintaining myocyte energy and cellular integrity only during ischemia or hypoxia [42]. Oxidative phosphorylation in the mitochondria is the primary mode of ATP generation in these cells, accounting for approximately 95% of the heart’s ATP production [43]. However, the reliance on aerobic metabolism, coupled with the fact that the myocardium is one of the most metabolically active tissues, comes with challenges. Reactive oxygen species (ROS), primarily generated as byproducts of the electron transport chain in the mitochondria, can accumulate under metabolic imbalance or stress conditions. ROS can lead to oxidative injuries, which have been implicated in the neurodegeneration seen in Alzheimer’s and Parkinson’s disease and in cancer, diabetes, and cardiovascular diseases. ROS may also have non-deleterious effects, functioning in regulating intracellular signaling pathways, redox modifications of proteins, and as intracellular messengers. Since ROS has a role in physiological and pathological conditions, properly regulating ROS homeostasis is crucial.

In the context of human pancreatic cancer cells, intriguing insights into the promotion of ferroptosis have emerged, particularly involving the endoplasmic reticulum protein stimulator of interferon genes (STING1) [44]. It has been observed that STING1 accumulates within mitochondria, where it engages with the mitochondrial outer membrane proteins mitofusins (MFN1/2) [44]. This interaction instigates mitochondrial fusion, leading to the generation of reactive oxygen species (ROS) and subsequent lipid peroxidation, ultimately culminating in the initiation of ferroptosis [44]. Studies employing both in vitro models and xenografted mouse experiments have highlighted the pivotal role of STING1 and MFN1/2 genes in regulating the sensitivity of pancreatic cancer cells to ferroptosis [45]. Within the intricate landscape of cellular processes, ROS, primarily derived from mitochondrial metabolism, emerge as critical mediators in intracellular signal transduction [1]. Key ROS species, such as hydrogen peroxide (H_2_O_2_), superoxide anion, hydroxyl radical, and peroxynitrite, play a central role in rendering cells susceptible to ferroptosis [46]. Mitochondrial respiration-induced ROS have been shown to damage enzymes in the mitochondrial respiratory chain complex. Notably, inhibition of SLC7A11 or cystine deprivation results in reduced glutathione (GSH), impairing its ability to clear ROS. Additionally, SLC7A11 inactivation leads to the accumulation of glutamate, subsequently enhancing tricarboxylic acid (TCA) cycling, causing hyperpolarization of the mitochondrial membrane potential, and ultimately promoting ferroptosis [47,48].

The interplay of hydrogen peroxide and unsaturated fatty acids containing Fe^2+^ in the Fenton reaction leads to the formation of polyunsaturated fatty acid free radicals [1]. These radicals, reacting with oxygen, generate polyunsaturated fatty acids peroxygen free radicals, triggering cell death. Noteworthy changes in mitochondrial TCA cycling and glycolysis further contribute to the promotion of ferroptosis, as heightened TCA cycling activity increases cellular sensitivity to ferroptosis [1]. Key enzymes within the TCA cycle, such as the α-ketoglutarate dehydrogenase complex (KGDHC), regulate ferroptosis, with its inactivation inhibiting the occurrence of this cell death pathway [1]. The Fenton reaction, facilitated by iron ions, generates hydroxyl free radicals from hydrogen peroxide, inducing lipid peroxidation [49]. In the absence of mitochondrial DNA, cells exhibit elevated levels of Fe^2+^, resulting in the production of hydroxyl radicals from hydrogen peroxide. Aquaporins (AQP) 3, 5, and 8 have been identified to bind to nicotinamide adenine dinucleotide phosphate oxidase 2 (NOX2), regulating the permeability of extracellular hydrogen peroxide and thereby contributing to the occurrence of ferroptosis [50]. These intricate mechanisms underscore the multifaceted nature of ferroptosis regulation within the intricate cellular milieu.

Mitochondria play a crucial protective role against ferroptosis through the regulation of Coenzyme Q (CoQ) synthesis and transport [44]. The lipid transporter StAR-related lipid transfer domain protein 7 (STARD7) is a key player in this process, being essential for both CoQ synthesis within mitochondria and its transportation to the cell membrane [51]. Within mitochondria, STARD7 safeguards oxidative phosphorylation and the morphogenesis of mitochondrial ridges [44]. Simultaneously, in the cytoplasm, STARD7 facilitates the transport of CoQ to the cell membrane, preventing iron oxidation. Intriguingly, an increase in STARD7 expression in the cytoplasm enhances cellular resistance to ferroptosis [52]. Another enzyme, glycerol-3-phosphate dehydrogenase 2 (GPD2), contributes significantly to the protection against ferroptosis by inhibiting mitochondrial lipid peroxidation. GPD2 generates reduced CoQ-H2 in the inner mitochondrial membrane, thus acting as a defense against cellular ferroptosis. Metabolome analysis has uncovered that the treatment with GPX4 inhibitors results in the depletion of glycerol-3-phosphate (G3P) in cells. Subsequent investigations demonstrated that supplementing cancer cells with G3P attenuates ferroptosis induced by GPX4 inhibitors in a GPD2-dependent manner. The deletion of GPD2 in cancer cells heightens their sensitivity to mitochondrial lipid peroxidation and ferroptosis caused by GPX4 inhibition. Notably, the simultaneous knockdown of GPX4 and GPD2 synergistically inhibits tumor growth driven by ferroptosis [53]. These findings underscore the mechanisms orchestrated by mitochondria and associated enzymes in fortifying cellular resilience against ferroptosis.

Iron is a potentially catastrophic substrate for generating ROS in the heart. Iron uptake in cardiac myocytes primarily occurs through the transferrin-mediated pathway. Iron-loaded transferrin binds to the transferrin receptor (TFR1) on the cell surface. This binding facilitates endocytosis of the holo-transferrin-TFR1 complex. Iron dissociates from transferrin inside the endosomes and is converted from its ferric (Fe^3+^) to ferrous (Fe^2+^) form by STEAP metalloreductases. Divalent metal transporter 1 (DMT1, also known as NRAMP2) helps transport this iron to the cytoplasm [54]. Within the cytoplasm, iron serves critical functions. It is a vital cofactor for various enzymes and essential for heme synthesis. Excess iron, not immediately utilized, is stored in the ferritin complex to prevent cytotoxicity. Ferritin synthesis surges when intracellular iron levels rise.

Moreover, iron can be mobilized from ferritin stores through ferritinophagy, where the autophagy machinery targets ferritin for lysosomal degradation, releasing its stored iron [55]. Ferroportin serves as the primary membrane transporter responsible for exporting iron from the inside of a cell to its external environment. Positioned on the cell membrane, it facilitates the movement of intracellular iron to the extracellular space. Once the iron is outside the cell, it binds to transferrin, a primary iron transporter in the bloodstream. The expression and activity of ferroportin are controlled by hepcidin [56]. Produced predominantly in the liver, hepcidin acts as a negative regulator of iron transport by binding to ferroportin. This binding initiates the internalization and degradation of ferroportin, consequently reducing iron efflux from the cell.

Genetic hepcidin deficiency can lead to severe forms of hemochromatosis and, consequently, abnormally high iron levels in the heart [57]. Many other genetic causes may lead to iron overload in the heart. Specific mutations in the gene encoding for ferroportin can prompt a form of hereditary hemochromatosis typified by excessive iron accumulation in tissues. In a cardiac-specific context, any alteration that impairs the function of ferroportin can lead to increased cardiac iron, ultimately impairing heart function. Studies involving mice have demonstrated that cardiomyocyte-specific deletions of ferroportin result in notable iron overload in the heart [58]. The consequences of iron overload can be highly deleterious for the heart. Intracellular iron can catalyze polyunsaturated fatty acids’ peroxidation in cardiac myocytes’ lipid membranes. In the presence of ROS, especially in oxidative stress conditions, these fatty acids undergo structural changes, producing lipid hydroperoxides. The accumulation of lipid hydroperoxides, in the absence of efficient repair mechanisms, can trigger ferroptosis, emphasizing the necessity of an efficient system to counteract the peroxidation effects [1].

Several regulatory levels of ferroptosis have been discovered in recent years. Those include Xc/GSH/GPX4, NAD(P)H/FSP1/CoQ10, and GCH1/BH4/DHFR. Out of these three systems, the Xc/GSH/GPX4 pathway is widely regarded as the key regular of the ferroptosis mechanism [59]. Glutathione peroxidases (GPXs) are a family of oxidoreductases observed in all living organisms and are considered a central component of the cellular antioxidant defense system. The majority of the mammalian GPX family are selenoproteins. Selenoproteins have strong antioxidant activity and are characterized by the presence of the 21st amino acid, selenocysteine. The presence of selenium tightly regulates the production of these enzymes. Although most GPXs reduce only small organic hydroperoxides, GPX4 is unique because it can reduce large and complex hydroperoxides and cholesterols, even those embedded in biological membranes. As such, GPX4 is the major player in protecting cardiac cells from oxidative damage. In the first phase of its action, the active site selenol (Se-H) of GPX4 is oxidized to selenic acid (Se-OH). This, in turn, reduces the toxic lipid hydroperoxides to their corresponding alcohols and free hydrogen peroxide to water [59]. During the second phase, a reducing substrate is needed to convert the selenic acid back to active selenol, thus closing the catalytic cycle and allowing the process to be repeated.

GPX4 is unique from the rest of the GPX family because it is not limited to GSH as a reductant and may use other thiol-containing proteins as reductants. However, GSH is the most abundant reductant in cells. GSH regeneration is crucial; this is orchestrated by glutathione reductase, which converts GSSG back to GSH at the expense of NADPH, thus ensuring sustained GPX4 activity [60]. System xc- is also invariably linked to GSH and GPX4 activity, which function as an antiporter primarily facilitating the exchange of extracellular cystine (a dimer of two cysteine molecules linked by a disulfide bond) for intracellular glutamate in a 1:1 ratio. This process is critical for maintaining intracellular cysteine levels, which is rate-limiting for synthesizing GSH. After being transported into the cell, cellular reductants rapidly reduce cystine to two cysteine molecules. Cysteine’s role does not stop at being a GSH precursor; it also acts as an antioxidant and plays a part in protein synthesis and other metabolic pathways. A functional system xc- ensures a steady intracellular supply of cysteine, which is pivotal for GSH synthesis. Since GSH is a crucial cofactor for GPX4, a major anti-lipid peroxidation enzyme, the implications of a compromised system xc- go beyond just an imbalance in redox status. Insufficient GSH can hamper the cell’s ability to prevent the ferroptotic cascade initiated by unchecked lipid peroxidation. Apart from its role in GPX4-mediated lipid repair, GSH directly scavenges ROS and also aids in the regeneration of other antioxidants, such as vitamins C and E. Hence, a well-functioning system xc- indirectly fortifies multiple tiers of the cellular antioxidant defense [61]. The oncogenic RAS-selective lethal small-molecule Erastin, which functionally inhibits the cystine–glutamate antiporter system, accelerates ROS accumulation in ferroptosis [62].

The dysregulation of the aforementioned protective antioxidant mechanisms and subsequent increase in ferroptotic activity have been linked to several cardiomyopathies. Doxorubicin (DOX) is an essential chemotherapeutic drug. However, it is also linked to the severe side effect of cardiotoxicity, leading to a specific type of chronic, progressive, and fatal cardiomyopathy, termed DOX-induced cardiomyopathy (DIC). The molecular underpinning of DIC has been explored in various studies, revealing a complex interplay of mechanisms such as transcriptional misregulation via topoisomerase IIβ (Top2b) inhibition, abnormalities in calcium handling, iron accumulation in mitochondria, and mitochondrial dysfunction [62]. Notably, mitochondrial-derived oxidative stress emerges as a plausible root cause. This hypothesis is further supported by evidence showing that overexpressing antioxidative enzymes like manganese superoxide dismutase (Mn-SOD) and glutathione peroxidase1 (GPx1) can mitigate the effects of DIC. A critical ferroptosis regulator is previously touched upon GPX4, an endogenous scavenger for lipid peroxides (LPs). The importance of GPX4 in ferroptosis regulation has been previously evidenced by the fact that most ferroptosis inducers inhibit GPX4 either directly or indirectly, and inhibitors of ferroptosis are typically suppressors of lipid peroxidation, including radical-trapping antioxidants [63,64].

Research in this area led to the hypothesis that the dysregulation of ferroptosis and GPX4 under DOX treatment may contribute to LP accumulation and the progression of DIC. In a specific study by Tadokoro et al., this hypothesis was tested using GPX4 transgenic and heterozygous knockout (hetKO) mice [65]. A vital observation was that the downregulation of GPX4 and increased LPs cause cardiac impairment in DIC mice. A specific protocol was used to induce DIC in mice using DOX, which impaired left ventricular ejection fraction (LVEF). Furthermore, reduced heart weight and LV weight manifested as myocardial atrophy. After initial DOX treatment, a remarkable total and mitochondrial GPX4 downregulation was observed at the mRNA and protein levels, leading to increased lipid peroxidation. Specifically, mitochondrial malondialdehyde and acrolein, representing markers of lipid peroxidation, were markedly increased, pointing to mitochondria as responsible for LP increase in response to DOX. Histological analysis further revealed an increase in interstitial fibrosis. TUNEL staining in vivo (used for cell death in tissue sections) indicated increased TUNEL+ cells, potentially marking ferroptosis. Flow cytometry analysis confirmed that nuclei in ferroptotic cells induced by silencing GPX4 by using siRNA can be stained via TUNEL staining and apoptotic cells induced by staurosporine [65]. Further studies focused on GPX4′s role in regulating DIC progression. Overexpression of GPX4 in transgenic mice ameliorated DOX-induced cardiac impairments and reduced LPs, fibrosis, and TUNEL+ cells. In contrast, heterodeletion of GPX4 worsened the DOX-induced impairments. These findings underscore GPX4 as a critical regulator of DIC progression and demonstrate ferroptosis’s central role in DIC progression [65].

Another cardiomyopathy that deserves investigation is caused by myocardial ischemia-reperfusion injury (MIRI). Recent research has brought ferroptosis under the spotlight for its role in MIRI. The reperfusion phase in MIRI is marked by an intense ROS (reactive oxygen species) generation. While ROS have physiological roles and are typically counteracted by antioxidant defenses, the sudden inundation of oxygen during reperfusion can lead to an ROS imbalance [66]. In tandem with available iron, ROS catalyze the polyunsaturated fatty acids’ peroxidation in cellular membranes. In rat models of MIRI, this results in membrane fluidity and functionality alterations, marking the initiation of ferroptosis-induced cell death. Molecular markers such as malondialdehyde (MDA) become discernible, signifying lipid peroxidation [67]. Endoplasmic reticulum stress (ERS) offers another layer to this intricate web. The ER plays a pivotal role in synthesizing, modifying, and folding proteins. However, in conditions like MIRI, the balance is disrupted, leading to the accrual of misfolded or unfolded proteins.

Persistent ERS may steer cells towards apoptosis, signaled by markers like C/EBP homologous protein (CHOP) [68]. Studies have found that ferroptosis can lead to ERS activation and subsequent apoptosis by inhibiting system xc- [69]. Finally, intertwined with these processes is cellular autophagy—a catabolic mechanism responsible for the turnover of damaged or redundant cellular components. Autophagy is activated during MIRI, with markers like LC3-II (an autophagosome-associated lipidated form of LC3) and p62/sequestosome-1 indicating its activity [70]. Although autophagy generally functions as a protective mechanism, especially under conditions of nutrient deprivation or stress, in MIRI, its role is ambivalent. Unchecked autophagy can result in unwarranted cellular degradation and cell death. Intriguingly, autophagy might also intersect with ferroptosis. At the same time, it could mitigate ferroptosis by disposing of oxidized components; it can also enhance lipid peroxidation and ferroptosis by generating metabolic intermediates or affecting iron homeostasis [71].

Further investigation into myocardial ischemia-reperfusion injury (MIRI) has revealed that diabetes can exacerbate the damage incurred during reperfusion. In diabetes, elevated glucose levels and metabolic irregularities activate Nox2, a component of the NADPH oxidase complex responsible for generating reactive oxygen species (ROS) [72]. Conversely, AMP-activated protein kinase (AMPK), a serine/threonine protein kinase acting as a cellular energy sensor, is typically regarded as a protective kinase in the heart. When activated in response to low energy levels, AMPK initiates metabolic adjustments to restore energy balance and reduce oxidative stress. Diabetes-induced alterations in AMPK signaling may compromise the phosphorylation of its targets, disrupting cellular energy balance and exacerbating oxidative stress [73].

Pathological AMPK signaling in diabetes can further promote oxidative stress by activating Nox2. Under normal conditions, AMPK activation inhibits Nox2, thereby reducing ROS production. However, in diabetes, impaired AMPK signaling may lead to sustained Nox2 activation, contributing to increased oxidative stress and, consequently, elevated levels of ferroptosis, particularly within the myocardium during ischemia/reperfusion injury (I/RI) [74]. Notably, patients with diabetes face an increased risk of developing myocardial dysfunction, independent of reperfusion injury, coronary artery disease, or hypertension—a phenomenon known as diabetic cardiomyopathy [75]. Additionally, in 2022, ferroptosis was initially reported in the hearts of diabetic mice, with NRF2 activation demonstrated to prevent ferroptosis [76].

The connection between ferroptosis and NRF2 is crucial in atherosclerosis (AS), a complex disease characterized by smooth muscle proliferation and endothelial dysfunction [77]. The interplay among endothelial cells, smooth muscle cells, and macrophages plays a vital role in initiating and driving the AS process. Cholesterol-rich low-density lipoprotein (LDL) deposits in the vascular wall undergo oxidation to oxidized LDL under oxidative stress, leading to endothelial cell damage and immune responses. The NRF2-Keap1 pathway, a significant player in AS development, regulates ferroptosis, an iron-dependent form of cell death, and lipid peroxidation. NRF2 protects against oxidative stress and controls iron homeostasis through processes involving heme degradation by HO-1, the stress-inducible enzyme, and manipulation of cellular iron content through proteins like ABCB6 and FECH [78]. NRF2′s interaction with elements such as Tfr1, FTH1, FTL, and FPN maintains iron homeostasis, protecting cells from ferroptosis and influencing AS development [79].

P53, recognized as a tumor suppressor, actively promotes ferroptosis in AS by inhibiting glutathione (GSH) synthesis, thereby increasing cellular susceptibility to ferroptosis through elevated ROS levels. Additionally, p53′s modulation of enzymes like ALOX15, involved in fatty acid metabolism, promotes lipid peroxidation and ferroptosis, accelerating AS progression [80].

Another study of chronic kidney disease (CKD) using rat models provided further weight to the argument that ferroptosis plays a role in AS. The investigation reveals that ferroptosis is vital during calcium/phosphate-induced vascular calcification. They observed that the inhibition of ferroptosis significantly diminished calcification induced by high calcium and phosphorus in vascular smooth muscle cells (VSMCs) and arterial rings. From a mechanistic standpoint, suppressing the SLC7A11/GSH/GPX4 signaling pathway emerged as the key to the pro-calcific effect of ferroptosis in CKD [81]. Ferroptosis has also been implicated in sepsis-induced cardiac injury, hypertrophic cardiac conditions, and possibly cardiac arrhythmia [82].

## 4. Current and Future Treatment Options for CVD and Cardiomyopathy, including Using GSH and Iron Chelating Agents

Cardiovascular disease is a pressing health crisis in the United States, with more than 2200 Americans losing their lives to a form of CVD daily. It is consistently classified as the leading cause of death in the United States [83]. Due to the significant morbidity and mortality of CVD in the United States, there continues to be ongoing research on treatment options for patients who are diagnosed with a form of CVD or cardiomyopathy. According to current AHA guidance, treatment for cardiomyopathy is based on the underlying disease, with most patients having heart failure. Current ACC heart failure guidelines include lifestyle changes of reducing alcohol consumption, smoking cessation, exercise, and a low-sodium diet [84]. Pharmacological interventions include the administration of an angiotensin-converting enzyme (ACE) inhibitor or angiotensin receptor blocker, a loop diuretic, a beta-blocker, and spironolactone if the patient has class III or IV heart failure [85].

Current research is investigating therapeutic strategies that aim to counteract the oxidizing effect of reactive oxygen species, including reduced GSH [86]. GSH is a tripeptide found in all tissues and synthesized in the heart and the liver. Specifically, it has several functions in cells that contribute to cellular homeostasis but primarily functions as an antioxidant, abundant in the cellular environment [83]. Additionally, particular proteins may protect against H_2_O_2_ by increasing levels of GSH. Specifically, heat shock proteins 27 and 25 in humans and mice have been shown to increase the level of GSH [87]. Ongoing investigations have suggested that enhancing the GSH system and preventing ferroptosis, a type of cell death closely related to the GSH system, is a promising therapeutic strategy for several cardiac diseases [88]. Two strategies for enhancing the GSH system have been discussed in the literature. First, the direct administration of GSH in vivo has been shown to increase GSH and reduce oxidative stress markers [89]. However, this strategy is uncommon in myocardial injuries. Another approach under investigation is supplementing the precursors of GSH, including cysteine, glycine, glutamate, and selenium, to increase GSH levels in the myocardium [88]. Natural monomers may be of therapeutic benefit to patients with CVD by activating the GSH system and reducing oxidative stress on the heart through various mechanisms. These natural monomers discussed in the literature include terpenoids, phenolic acids, flavonoids, quinones, and alkaloids [88].

Quercetin, a flavonoid found in many fruits and vegetables, has been proven to improve serum total antioxidant capacity (TAC) in patients with post-myocardial infarction compared to the placebo group in a double-blinded, randomized clinical trial [90]. Further studies have demonstrated that adding quercetin to standard therapy improved systolic and diastolic cardiac functions and reduced the number of ST-segment depressions in patients with coronary heart disease [91]. These studies demonstrate that quercetin is a promising natural monomer in treating cardiac injury by activating the GSH system and reducing oxidative stress biomarkers in the heart [88]. Terpenoids or carotenoids are also natural compounds currently being investigated for therapeutic benefit in cardiac conditions; however, research findings thus far have been contradictory. Preliminary research has illustrated that β-carotene, a dietary carotenoid, can reduce oxidative stress and maintain myocardial GSH levels in rats [92]. However, more recent clinical studies have demonstrated that β-carotene supplementation may increase CVD risk and mortality [93]. Thus, further research on this compound must be performed prior to any clinical implementation in patients with cardiac disease. Additionally, phenolic acids are known to have significant antioxidant properties and health benefits in patients with CVD. For example, ferulic acid has been proven to decrease lipid peroxidation and prevent cardiac ferroptosis mediated by ischemia-reperfusion injury [94]. The State Drug Administration of China has recently approved a derivative of ferulic acid known as sodium ferulate for treating ischemic cardiovascular disease [95]. Although these natural monomers have been demonstrated to have therapeutic benefits in patients with different forms of CVD, challenges regarding bioavailability and biotoxicity remain under investigation, as longitudinal studies must be performed to determine whether these compounds have adverse effects on peripheral organs [89] (Table 1).

In addition to GSH system activators, iron chelating agents may also provide therapeutic benefits in patients with excessive free radical production secondary to iron toxicity. This excessive free radical production is associated with cardiac dysfunctions, including arrhythmia and cardiomyopathy [96]. The FDA has approved three primary iron chelators for clinical practice: deferoxamine, deferiprone, and deferasirox [96]. Although all of these iron chelators have the same primary purpose of prevention and elimination of iron overload, there are advantages and disadvantages associated with each agent [96]. Deferoxamine (DFO) is one of the first iron chelators approved to treat excess iron. However, this drug has low oral absorption and thus poses a significant limitation for clinical administration [97]. Adverse effects caused by DFO include growth retardation, skin reactions, allergic reactions, and bone abnormalities [96]. It has also been shown to have less of a cardioprotective effect than deferiprone (DFP) and thus is primarily used in thalassemia patients [98]. For various reasons, deferiprone (DFP) is critically important in treating cardiomyopathy and heart failure. It is used clinically as an oral iron chelator [96]. First, it reduces red blood cell membrane oxidative damage and the formation of oxidation products [99]. Further, it effectively eliminates excess iron from the heart and has been proven to improve left- and right-ventricular ejection fractions more efficiently than DFO [100]. Mortality and morbidity rates from cardiac events were also lower in patients who took DFP rather than DFO [101]. Deferasirox (DFX) was approved as an oral iron chelator for clinical use by the FDA in 2005 [101]. However, it is mainly used for thalassemia and the prevention of endocrinopathy [96]. This iron chelator is unique as it can improve bone mineral density and decrease the prevalence of osteoporosis in patients who take it after five years of treatment; however, minimal research has investigated its effect on cardiomyopathy and heart failure [96].

Future research aims to investigate the mechanism of iron chelation therapy and its cardioprotective effects. Additionally, combined therapy with multiple chelators remains a topic of future investigation as it may therapeutically benefit patients who do not improve with monotherapy. Future studies aim to determine the benefits of combination therapy in patients with CVD and any potential adverse reactions to better treat patients with CVD and cardiomyopathy [97].

## 5. Conclusions

CVD consistently maintains its position as the primary cause of mortality in the United States, displaying significant morbidity and mortality rates despite ongoing pharmaceutical interventions. This enduring prevalence underscores the imperative need for a deeper exploration of treatment modalities. Current approaches primarily revolve around addressing the patient’s underlying conditions, particularly heart failure, yet these have proven ineffective in stemming the relentless tide of CVD. The emerging concept of ferroptosis, a process implicated in CVD, has prompted investigations into ferroptosis inhibition as a prospective avenue for treatment. Recent clinical studies have provided support for employing glutathione in preventive strategies and iron chelation therapy for the treatment of CVD and cardiomyopathy induced by ferroptosis, with demonstrably positive outcomes. However, there remains a considerable gap in comprehending the full spectrum of prevention, treatment, and prognosis related to ferroptosis-induced CVD. Thorough and extensive research endeavors are essential to unravel the intricacies surrounding this phenomenon, thereby enabling a more comprehensive understanding and development of targeted therapeutic interventions.

## Figures and Tables

**Table 1:** Summary of GSH utility in cardiovascular disease.

Therapeutic Strategies for GSH Enhancement in CVD	Key Findings
GSH System Enhancement Strategies	Current research is exploring therapeutic strategies targeting reactive oxygen species, including reduced GSH, as a potential treatment for cardiovascular diseases (CVD) [71]. GSH is a tripeptide synthesized in the heart and liver, serving as a crucial antioxidant in cellular homeostasis [68]. Heat shock proteins 27 and 25 have been identified to elevate GSH levels and protect against H_2_O_2_ [72].
Prevention of Ferroptosls	Ongoing investigations suggest preventing ferroptosis, closely related to the GSH system, as a promising therapeutic approach for cardiac diseases [73]. Two strategles involve direct GSH administration and supplementing GSH precursors like cysteine, glycine, glutamate, and selenium [73].
Natural Monomers and GSH Activation	Various natural monomers, including terpenoids, phenolic acids, flavonoids, quinones, and alkaloids, activate the GSH system and reduce oxidative stress on the heart [73]. Quercetin, a flavonoid, demonstrated improved antioxidant capacity and cardiac function in post-myocardial infarction patients [75]. Ferulic acid, a phenolic acid, decreased lipid peroxidation and prevented cardiac ferroptosis in ischemia- reperfusion injury [79]. However, challenges related to bioavailability and biotoxicity warrant further investigation [74].
Terpenoids and Carotenoids	Preliminary research on -carotene, a dietary carotenoid, suggests its potential to reduce oxidative stress and maintain myocardial GSH levels in rats [77]. However, conflicting clinical studies indicate the need for further research before clinical implementation due to potential increased CVD risk and mortality [78].
Challenges and Future Research	Despite demonstrated therapeutic benefits, challenges such as bioavailability and biotoxicity remain under investigation, emphasizing the need for longitudinal studies to assess potential adverse effects on peripheral organs [74].
Clinical Applications	Ferulic acid, specifically its derivative sodium ferulate, has received approval in China for treating ischemic cardiovascular disease, highlighting the potential translational impact of these natural monomers [80].

## Data Availability

Data available in references.
